# Mechanics and Morphological Compensation Strategy for Trimmed Soft Whisker Sensor

**DOI:** 10.1089/soro.2020.0056

**Published:** 2022-02-14

**Authors:** Nhan Huu Nguyen, Van Anh Ho

**Affiliations:** School of Materials Science, Japan Advanced Institute of Science and Technology (JAIST), Nomi, Japan.

**Keywords:** morphological compensation, trimmed artificial whisker sensor, damaged soft structure

## Abstract

Recent studies have been inspired by natural whiskers for a proposal of tactile sensing system to augment the sensory ability of autonomous robots. In this study, we propose a novel artificial soft whisker sensor that is not only flexible but also adapts and compensates for being trimmed or broken during operation. In this morphological compensation designed from an analytical model of the whisker, our sensing device actively adjusts its morphology to regain sensitivity close to that of its original form (before being broken). To serve this purpose, the body of the whisker comprises a silicon-rubber truncated cone with an air chamber inside as the medulla layer, which is inflated to achieve rigidity. A small strain gauge is attached to the outer wall of the chamber for recording strain variation upon contact of the whisker. The chamber wall is reinforced by two inextensible nylon fibers wound around it to ensure that morphology change occurs only in the measuring direction of the strain gauge by compressing or releasing pressurized air contained in the chamber. We investigated an analytical model for the regulation of whisker sensitivity by changing the chamber morphology. Experimental results showed good agreement with the numerical results of performance by an intact whisker in normal mode, as well as in compensation mode. Finally, adaptive functionality was tested in two separate scenarios for thorough evaluation: (1) A short whisker (65 mm) compensating for a longer one (70 mm), combined with a special case (self-compensation), and (2) *vice versa*. Preliminary results showed good feasibility of the idea and efficiency of the analytical model in the compensation process, in which the compensator in the typical scenario performed with 20.385% average compensation error. Implementation of the concept in the present study fulfills the concept of morphological computation in soft robotics and paves the way toward accomplishment of an active sensing system that overcomes a critical event (broken whisker) based on optimized morphological compensation.

## Introduction

Due to frequent burrowing and inhabiting in dark narrow spaces, rats elaborately control their muscular whiskers to make rhythmic back-and-forth swing for environment exploration.^1^ Whisker-obstacle contacts cause the whisker to bend stimulating mechanoreceptive neurons in follicle complex.^2^ Many scientists in biology and psychology have been inspired by how rodents utilize tactile sensation to accomplish tasks, including localization^3^ and texture discrimination.^4^ In addition, constructing a biomimetic whisker-based sensor could facilitate not only research of natural behavior in rodent sensing but also a proposal of bioinspired solutions for sensing capability in mobile robots, especially ones operating in complex and dangerous terrains that are inaccessible for humans such as collapsed tunnel, building or rooms filled with gas or smoke, and so on.

**FIG. 1.**
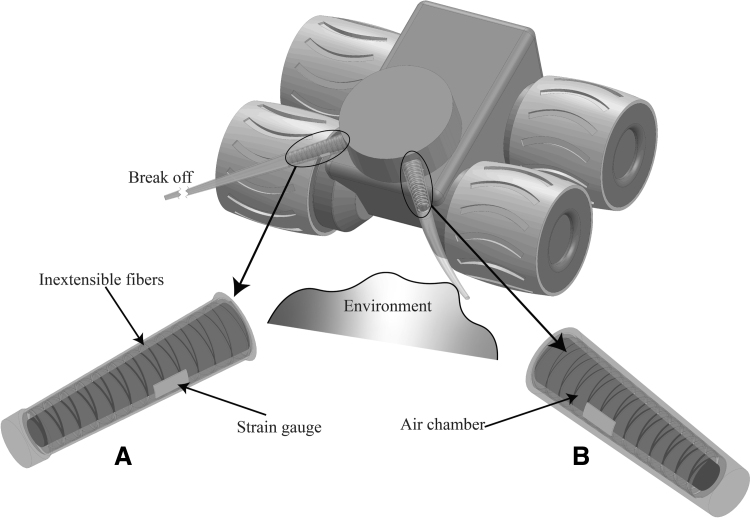
Illustration of the proposed whisker sensory system that was integrated into a mobile robot. This whisker can actively change the morphology of the integrated chamber upon pressurization to adapt to the critical circumstance of its morphology (being trimmed, broken, or partly damaged) during operation. **(A)** The integrated chamber is pressurized at a predetermined value in compensation mode, when the whisker was broken. **(B)** Chamber in normal mode (without any damage).

Recently, autonomous robots have been a focus of the engineering and research community for their ability to operate independently in unstable and hazardous conditions.^5^ To achieve this ability, sensory systems of robots require both competencies in sensing ability and flexibility in responding to contingencies. However, adaptability of sensing components had not been considered as a crucial feature, especially for artificial vibrissa systems whose whisker tips are frequently eroded, trimmed, or broken (Fig. 1). To deal with such events (e.g., broken sensor whiskers), autonomous robots normally have to stop their operation, then be manually repaired or replace the broken sensing components. To overcome this limitation, effort must be paid to on-site solutions, especially development of control algorithms that can counter problems of broken components at high levels of uncertainty.^6^

In this article, we suggest an approach that utilizes a morphological computation technique, which can be referred in Pfeifer and Gómez,^7^ to offload the complexity of the control system and take over the adaptive function. Inspired by how rodents adjust the capacity arrangement of “barrels” in their primary somatosensory cortex in adaption to broken whisker, and the dependence of mechanical properties on the medulla layer of the whisker,^1^ we introduce an intriguing prototype of a soft artificial whisker sensor that can actively vary its sensitivity with change in chamber morphology (similar to the medulla layer), changing the chamber morphology, including geometrical and mechanical specs such as shape and softness, as illustrated in Figure 1A.

The ultimate goal is to enable autonomous robots to *maintain sensibility* despite a broken sensory system and remain functioning until the next maintenance. To support this idea, the morphology of the inner chamber of the whisker is actuated by pressurized air to change the tension of the outermost layer where a strain gauge is bonded as a receptor. Although there are no sensing receptors on the outer surface of a rodent's whisker, there are many reports of insect antennae with sensing components along their length.^8^ On top of that, as far as the authors know, no study has previously used the rodent ability to compensate for a trimmed bio-whisker in a robotic system. Our study demonstrates the advantage of soft materials over rigid materials for artificial whiskers and the importance of researching the influence of whisker morphology in tactile perception.

## Related Works

### Bioinspired active vibrissal sensor in robotics

Haptic sensing through *touch* offers enormous potential to robots in assessing their surrounding environment, including interaction with human beings. There has been a great deal of research regarding proposals of new tactile sensing systems based on a variety of touch phenomena. Vibrissa sensory systems are typical examples that were inspired by the tactile discriminatory abilities of natural animals.

The first implementation by Russell and Wijaya^9^ involved a continuum rigid wire to extract contact location, shape, and contour information regarding a target object through contact interaction. Tactile responses were collected by a simple mechanical system comprising a servo-potentiometer and springs. In the following years, a series of studies focused on the application of biomimetic whiskers in robotics. Kaneko *et al.*^10^ established the first idea of investigating the correlation between the curvature of a flexible wire and tactile information. The term *rotational compliance* introduced in this work was also used as a fundamental hypothesis to explain how rats convert perceptible mechanical signals received during the sensing process into perceivable tactile information.^11^ Ahn and Kim^12^ proposed another example of a bio-whisker system integrated into the Koala robot platform. A Hall-effect magnetic sensor was used to measure the protraction angle to detect contact location, as well as the texture of the object. Researchers in Emnett *et al.*^2^ designed an artificial follicle containing four pairs of strain gauges that functioned as mechanoreceptors at the follicle-sinus complex. By collecting and analyzing a set of three different mechanical components related to bending moments and axial force along the length of the whisker, the location of the whisker-object contact point in three-dimension working space could be determined. Anderson *et al.*^13^ suggested an efficient solution to the problem of discriminating sensory responses caused by self-active movement based on the SCRATCHbot robotic platform. More recently, TacWhiskers were proposed in Lepora *et al.*,^14^ in which a vision-based vibrissal sensor utilizes a camera to capture the deformation of inner nodular pins upon interactions with obstacles. Besides inspiration from land mammals, systems inspired by whiskers of aquatic animals have been proposed such as flow-whisker sensor.^15^ One system mimicked the seal's hunting ability of using its whiskers to track vortices left by moving prey.^16^ A similar effort inspired by seal whiskers was introduced by Zhu *et al.*,^17^ which was able to work on the land (exploring an obstacle and its surface), as well as underwater (detecting water flow direction and velocity).

In general, most of these whisker sensors focus on embedding sensing components into the base, similar to the follicle-sinus complex in mammals, where external impacts along the length are transferred to the base.^2,18^ Such models were considered efficient copies of rodent's natural behavior in environment exploration and were proven successful in performance of sensing and navigation tasks by robotic systems. Nevertheless, consideration of systems that can adapt to change the mechanical properties (elasticity, stiffness, moment of inertia) with variation in geometry of the whisker is still limited. The novelty of our study lies in the intriguing inspiration from bio-whisker's structure analysis and plasticity in the neural circuits to create advantageous adaptability for the artificial whisker sensory system.

### Morphological computation and soft active tactile sensing

Morphological computation is a method that exploits the structure, material characteristics, and dynamics of a flexible body during interaction with its environment and thus outsources relevant computational function to the “body” rather than assign it to “central nervous system.”^19^ This concept has been widely applied to the design of bioinspired robots and related applications, especially in soft robotics. Hauser *et al.*^20^ exploited static feedback generated from a muscle-skeleton system (equivalent to a mass-spring system) to generate periodic locomotion. Nakajima *et al.*^21^ proposed a soft arm inspired by the octopus and demonstrated that the high-dimensional nonlinearity system could be partially emulated using the dynamics of a physical body.

Recently, the demand for improving the interaction of robots with humans and the surrounding environments has been growing. Since soft-bodied robots are inherently soft and deformable, it is expected that they can not only efficiently accommodate their environment but also actively change their own morphology to realize various sensing tasks. For example, Ho *et al.*^22^ introduced a simple active sensing platform Wrin'Tac, which mimicked water-induced wrinkles on a human finger by extended periods in a wet environment. They argued that changing morphology of the contact surface would give benefit in varying the stimuli perceived from the environment and allow the agent to actively select the sensing modality. Similarly, Qi *et al.*,^23^ also inspired by human wrinkles, addressed the idea of forming wrinkles to localize the sliding movement on a soft path, as well as discriminate a surface profile. Further research highlighted the potential of using embodiment to explain the behavior of a robot^24^ and gave some clues to understand theoretically how an organ's morphology defines the agent's behavior.

Regarding the whisker sensor, most recent studies have used steel or aluminum wires to fabricate such devices. However, such materials may experience unexpected oscillation of the whole structure during strong external interactions. Moreover, there is little work considering the medulla layer, which plays an important role in the mechanical properties of the vibrissae,^25,26^ in designing robotic whiskers. In this study, we propose a design of soft whisker sensor with novel inclusion of the medulla layer, which plays a crucial role in variation of whisker sensitivity upon morphological change. The main contributions of this article can be summarized as follows:

(1) Proposal of a novel structure sensory system mimicking the geometry of a rodent whisker with the ability to compensate if broken/trimmed or eroded during operation.
(2) Construction of a detailed analytical model to describe and compute morphological deformation. Through this model, we can predict the required chamber morphology for the compensation process, namely a morphological compensation strategy.
(3) A suggestion of a new platform for soft tactile sensing systems that can actively change its morphology to facilitate desired responses.

## Idea and Fabrication of Soft Whisker Sensing System

### Idea

**FIG. 2.**
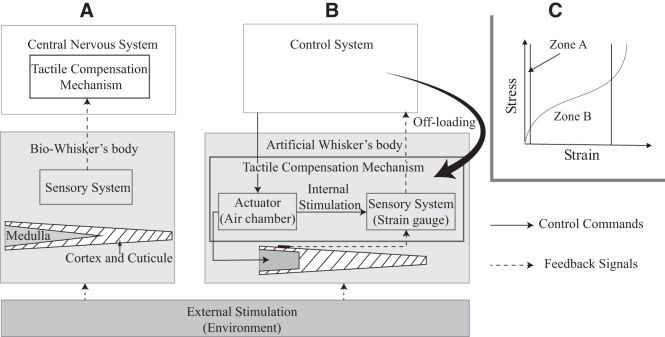
**(A)** An illustration diagram of how lost tactile information is compensated using a neuron compensation mechanism in rodent's brains^29^ in comparison with **(B)** the information flows of our morphology-based compensation method which assigns the compensation tasks to the whisker's body. **(C)** The typical stress–strain curve for silicon-rubber material.

Mammalian vibrissae are of interest not only to biologists but also to neuroscientists, thanks to their exquisite features. In this landscape, a question that arises is: How does a rodent cope when a whisker is trimmed? Many studies in biology and neuroscience have tackled this question and characterized the contribution of whiskers to sensorimotor development by trimming collective whiskers of rats and observing their behavioral adjustments,^27^ as well as the structural dynamics of neocortical neurons when those rats grew up.^28^ Holtmaat and Svoboda^29^ reported a noticed phenomenon which demonstrated how a rodent's central nervous system (i.e., *brain*) adapts to a broken whisker by adjusting neural region (known as the barrel cortex), where sensory signals are transferred from the snout so that the broken whisker (with decreased sensitivity) would be functionally replaced by its neighboring intact whiskers that appear to be more sensitive as *compensators*. In short, rodents compensation mechanism of tactile sensing is accomplished at the central nervous system as illustrated in Figure 2A.

Assuming that such mechanism was implemented in a robotic system, the control system integrated with data-based learning techniques should be exploited to address the scenario of losing tactile information due to broken structure. However, this implementation generally requests the controller to share its limited resources for computation tasks when the whisker was broken (Fig. 2A). To reduce such computation burden in biomimetic system, in this study, we approached differently by assigning the adaption process from the central system to the whisker's body (i.e., the chamber) itself as inspired by the proposal of *morphological computation.*^19^ More specifically, as demonstrated in Figure 2B, actively actuating the air chamber is expected to change not only the whisker's geometry but also its mechanical characteristic. By doing so, the sensitivity of the sensing element (i.e., strain gauge) could be controlled in the way of compensating for incorrect tactile signals during interaction with the surrounding environment. In addition, it allows the decentralization of the control system from the central (brain) to the local one (body), thus ideally reducing computation burden at central level.

In terms of the whisker body, the internal structure plays a critical role in transferring tactile signals to mechanoreceptors. According to Lucianna *et al.*,^1^ a bio-whisker has three separate layers: cortex, cuticle, and medulla (Fig. 2A, B), in which the medulla layer, which tapers from the base toward the tip, is the *softest* layer of the whisker. This suggests that a whisker could produce different levels of *elasticity* as reflected in *Young's modulus E* (explained in the next section) by reshaping the medulla layer, such that perceived signals induced by an external contact would be adjusted as expected due to the heterogeneous structure. The design of a proposed artificial whisker based on this hypothesis is introduced in the following sections.

Although the concept of “compensation” in nature varies among species, it is worthwhile investigating this function in a robotic device. As suggested by Solomon and Hartmann,^30^ the contact distance is generalized for any whisker by following equation: $Contactdistance=a\times L_{a}$, where *a* is the contact ratio. Based on this assumption, we propose a compensation concept: with the same contact ratio *a*, the tactile information perceived by intact whiskers in an array (either longer or shorter) is supposed to be as close to the broken one as possible. At the same time, for a sole whisker which requires a self-calibration procedure, compensation will be activated to obtain updated sensitivity (in the broken case) that is close to the original one. These cases will be discussed in Experimental Results section.

### Silicon-rubber characterization

Thanks to the softness of silicon rubber, the morphology of a soft structure is actively changed by the integration of a simple pneumatic actuator. It is worth noting that the response of a soft material to stress highly depends on the applied strain, and the stress–strain relationship may vary significantly as illustrated in Figure 2C. More importantly, the linear theory of elasticity is only valid for a very small deformation that corresponds to zone A of the stress–strain curve, which obeys Hooke's law, and is characterized by a constant Young's modulus. As stress increases slowly with high deformation in zone B, it is expected that the applied Young's modulus in this region reduces, which may result in a change of the soft body morphology. This characteristic is applied in our method to achieve the changeable morphology of an artificial whisker.

### Design and fabrication of artificial whisker

**FIG. 3.**
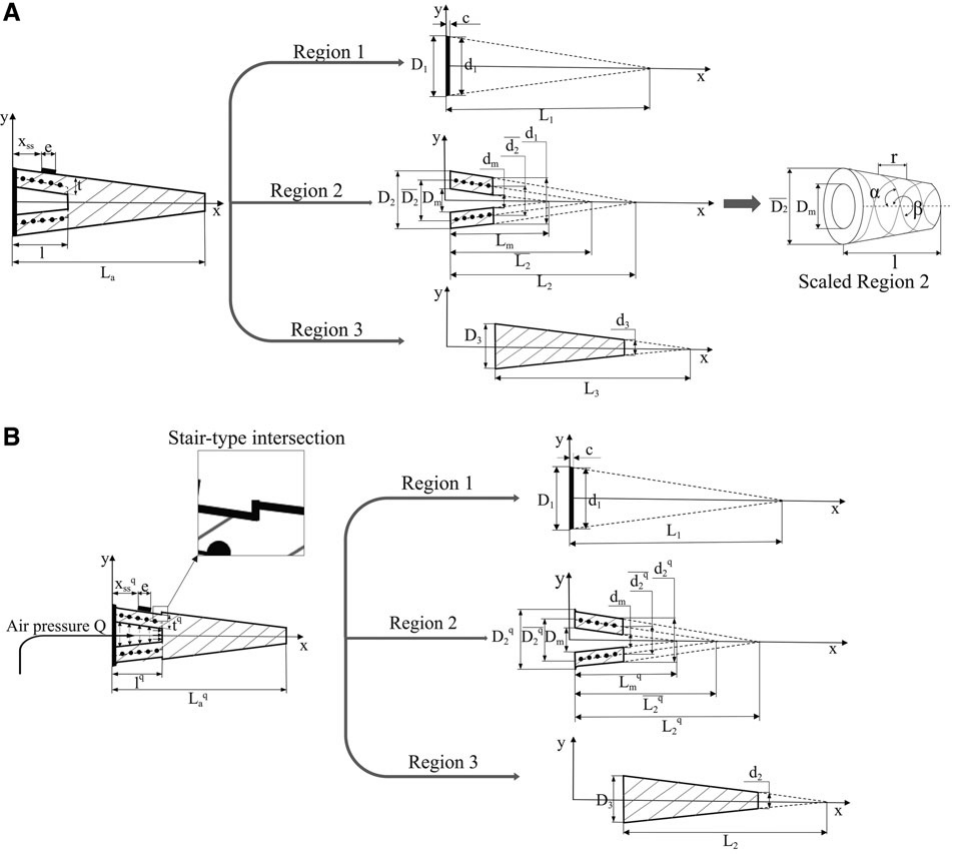
The design and dimensions of the artificial whisker for each region: **(A)** Before pressurization. **(B)** After pressurization. Note that the structure of the whisker after air pressurization process and most of the changed variables belong to the region 2. See Table 1 for details.

To achieve the soft changeable morphology in our device, we replicated the shape of a linearly-tapered natural whisker using silicon rubber and introduced a unique occurrence of the medulla chamber inside the whisker as illustrated in Figure 2B. The proposed whisker was divided into three separate regions to facilitate construction of the analytical model: cap (region 1), chamber (region 2), and whisker body (region 3) (Fig. 3). First, the chamber (region 2) is tapered at an equivalent degree of taper as the outer layer, that is, t(thickness) remains constant. Second, a thin cap (region 1) covers the chamber to prevent air leakage and to house a cylindrical hose that feeds pressurized air in or out. Third, a strain gauge is fixed to the top (outside wall) of the chamber with the sensitive direction toward *x*-axis. To prevent all unwanted influences of chamber expansion in other directions but *x*-direction to the strain gauge, two inextensible fibers (Nylon nano-filament of diameter ϕ=0.38 mm) are wrapped helically with angles α and β around the chamber as illustrated in Figure 3A. Bishop-Moser *et al.*^31^ suggested that α and β should be larger than 54.7° and 234.7°, respectively, to constrain mobility in all directions but the axial translation in *x*-direction. Thus, to meet this requirement, we designed the helical path to have the pitch r=3 mm. All essential parameters for the analytical model and visualization are presented in Table 1 and Figure 3, respectively.

**Table 1.** Artificial Whisker Parameters (Fig. 3)

Parameter	Value (mm)	Parameter	Value (mm)
*D*_1_	18	*L*_2_	178
$D_{2}=d_{1}$	17.8	*L*_3_	165
$D_{3}=d_{2}$	16.5	$\bar{L_{2}}$	148
$\bar{D_{2}}$	14.8	*L_m_*	120
*D_m_*	12	$x_{ss}$	6
$\bar{d_{2}}$	13.5	*c*	2
*d*_3_	11	*t*	3
*d_m_*	10.5	*l*	15
*L*_1_	180	*e*	2

**FIG. 4.**
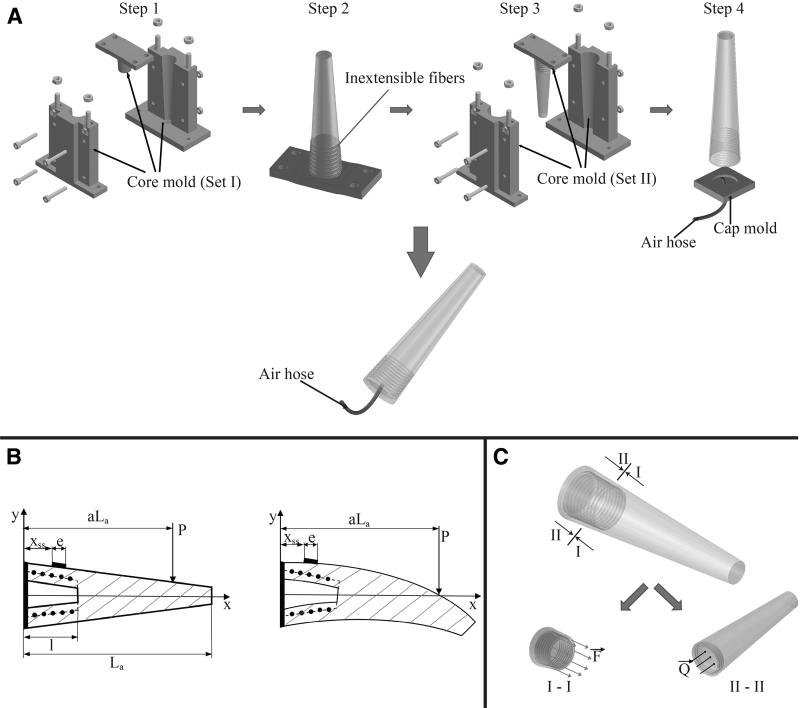
**(A)** Fabrication procedure consisting of four consecutive steps: Step 1: Mold the inner layer of the whisker body using the core molds set I. Step 2: remove the body's mold but leave the medulla mold to hold the shape of the chamber, then wrap slightly with the nylon fiber. Step 3: Coat an outer skin to keep the reinforced fiber in place with the core mold set II. Step 4: remove the body and the head mold then dip the big end into a thin layer of Dragon Skin 30 inside the combination of cap mold and an air hose. **(B)** The whisker encounters the contact force *P*, and then, the change in form of the whisker and the mechanical signal generated in the strain gauge will be analyzed. **(C)** Schematic analyzing the internal force exerted in each cross section along the length of region 2.

The fabrication procedure is summarized in Figure 4A. We used silicone-rubber Dragon Skin 00-30 (Smooth-on, Inc., PA) to make the soft whisker body. Molds were designed using 3D-CAD software (SolidWorks) and fabricated in a 3D printer (Zortrax M200; Zortrax, Olsztyn, Poland). Two different sets of core molds were used in sequence due to the complicated design. First, core molds of set I were used to form the double-helical trench to make ease for wrapping the inextensible fibers in the next step. Then, set II was used to obtain all the dimensions of the outer layer as designed in Table 1. Both sets shared the same medulla mold, which was not removed until finishing step 3. The model was then set on a cap mold with a cylindrical air hose (outer diameter 2 mm), and a thin layer of Dragon skin 30 was used to create the cap. Finally, a strain gauge was bonded by glue at the designate site $x_{ss}$ and covered by a thin layer of silicone glue to enhance its robustness against external loads. The strain gauge used in this article is KFGS-2-120-C1-11 L1M2R with gauge factor GF=2.21%±1%, grid length e=2 mm, and was bonded with adhesive glue CC-3A, both from Kyowa Electronic Instrument Co. (Japan). The fabrication procedure was completed by fitting the model to the base, and the curing process was implemented at a warm temperature with help from the vacuum dryer AVO-200NB-CR for getting rid of all possible bubbles.

## Analytical Model for Normal and Compensation Mode

As aforementioned, a typical whisker-like sensing system accomplished various tactile exploration tasks that happened on the whisker length. However, in the present study, we particularly focused on contact localization ability, that is, specifying where contact occurs along the body of the whisker. Therefore, we aimed to propose an analytical solution to tackle this issue, as well as a platform for a morphological compensation strategy. For the sake of simplicity, the following assumptions were made:

- All possible interactions will be assumed to exert only in region 3 of the whisker body to minimize estimation error due to high rotational stiffness. In practice, this hypothesis is biologically relevant to the fact that rats tend to make whisker-environment collisions near the tip to avoid the increasing spike rate of some cells in follicle when contact is close to the snout.^32^
- Lateral slip at the contact location is neglected.
- Deformation of the whisker is within the measurable range and principle measuring plane of the strain gauge.
- A single-point contact is solely considered.

### Normal mode

We attempted to construct a model for prediction of the strain gauge output upon contact with the external environment, which depends on the geometry, location of applied force, and deformation of the proposed whisker. Especially, the correlation between the morphology of the whisker model and the medulla chamber is of particular interest with an intrinsic effect on the response of the sensor system. Therefore, an effective analytical model was not only necessary to assess our idea but also applicable for operation in a robotic system.

When the whisker encounters an obstacle, the contact force exerted at a location along the body generates a bending moment as illustrated in Figure 4B. We focused on expressing the ratio of the interpreted tactile signal (or the mechanical strain) to whisker deflection at the contact point. This approach is one of two fundamentally different methods for performing tactile perception extraction used by Refs.^10,12^ However, such effort used classical beam theory Euler–Bernoulli, which is only applicable for a beam that has a *homogeneous* cross section and material property (Young's modulus). As a result, for our nonuniform whisker design, we used Castigliano's theorem^33^ that relates to strain energy generated at each section of the whisker body. In short, by assessing the strain energy stored in a soft structure during its deformation, the displacement of the contact point is identified. First, the principle of conservation of energy is given by:

(1) $$U=\sum W_{i}=\int_{0}^{aL_{a}}\frac{M{\left(x\right)}^{2}}{2E_{i}I_{i}\left(x\right)}dx,$$

where *U* denotes the strain energy, *W_i_* is total work done by internal forces, M(x) is bending moment internally generated in the whisker body, and $I_{i}\left(x\right)$ and *E_i_* (i=1,2,3) represent the second moment of cross-section area and Young's modulus for each region, respectively. The product *EI* is named flexural rigidity since it is a measure of bending resistance. $I_{1}\left(x\right)$, $I_{3}\left(x\right)$, and M(x) are calculated as following equations:

(2) $$I_{1}\left(x\right)=\frac{\pi {d_{1}}^{4}\left(x\right)}{64}=\frac{\pi {D_{1}}^{4}{\left(L_{1}-x\right)}^{4}}{64{L_{1}}^{4}},$$

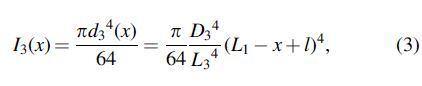


where $d_{i}\left(x\right)$ is the diameter of the cross-sectional area at position *x* from the base. In regards to the term $I_{2}\left(x\right)$, the expression for second moment of section area for region 2 is supposed to be: $I_{2}\left(x\right)=\frac{\pi \left({d_{2}}^{4}\left(x\right)-{d_{m}}^{4}\left(x\right)\right)}{64}$. Nevertheless, we realized that it is a challenge to solve Equation (1) with explicit output. To deal with this issue, an approximation approach by considering region 2 as a thin tube was applied. For instance, let us consider the big end of region 2, we assumed that $D_{2}\approx \bar{D_{2}}\approx D_{m}$ and $t=D_{2}-D_{m}$. The formulation of second moment of area for this cross section $I_{2}\left(x\right)$ is derived as follows:

(5) $$\begin{array}{cc} I_{2}\left(x\right)=\frac{\pi }{64}\left(D_{2}^{4}-D_{m}^{4}\right) & =\frac{\pi }{64}\left(D_{2}^{2}+D_{m}^{2}\right)\left(D_{2}+D_{m}\right)\left(D_{2}-D_{m}\right)\\ & \approx \frac{\pi }{8}{\bar{D_{2}}}^{3}t \end{array}$$

Then, the generalized formulation of $I_{2}\left(x\right)$ can be rewritten as:

(6) $$I_{2}\left(x\right)=\pi \frac{{\bar{d_{2}}}^{3}\left(x\right)}{8}t=\frac{\pi }{8}\frac{{\bar{D_{2}}}^{3}}{{\bar{L_{2}}}^{3}}{\left(\bar{L_{2}}-x+c\right)}^{3}t.$$

According to Castigliano's theorem, displacement $\delta_{n}\left(x\right)$ at the contact spot is equal to the partial derivative of the total strain energy with respect to contact force:

(7) $$\begin{array}{cc} \delta_{n}\left(aL_{a}\right)=\frac{\partial U}{\partial P} & =\int_{0}^{c}\frac{M\left(x\right)}{E_{1}I_{1}\left(x\right)}\frac{M\left(x\right)}{\partial P}dx+\int_{c}^{l}\frac{M\left(x\right)}{E_{2}I_{2}\left(x\right)}\frac{M\left(x\right)}{\partial P}dx\\ & +\int_{l}^{aL_{a}}\frac{M\left(x\right)}{E_{3}I_{3}\left(x\right)}\frac{M\left(x\right)}{\partial P}dx \end{array}$$

In normal mode, Young's modulus is constant for the whole whisker's body $E_{1}=E_{2}=E_{3}=E$. Then, we get:

(8) $$\begin{array}{cc} \Leftrightarrow \delta_{n}\left(aL_{a}\right) & =\frac{64P{L_{1}}^{4}}{E\pi {D_{1}}^{4}}\int_{0}^{c}\frac{{\left(aL_{a}-x\right)}^{2}}{{\left(L_{1}-x\right)}^{4}}dx\\ & +\frac{8P{\bar{L_{2}}}^{3}}{E\pi t{\bar{D_{2}}}^{3}}\int_{c}^{l}\frac{{\left(aL_{a}-x\right)}^{2}}{{\left(\bar{L_{2}}-x+c\right)}^{3}}dx\\ & +\frac{64P{L_{3}}^{4}}{E\pi {D_{3}}^{4}}\int_{l}^{aL_{a}}\frac{{\left(aL_{a}-x\right)}^{2}}{{\left(L_{3}-x+l\right)}^{4}}dx \end{array}$$

(9) $$\delta_{n}\left(aL_{a}\right)=\frac{P}{E\pi }\left(\delta_{1}+\delta_{2}+\delta_{3}\right),$$

where


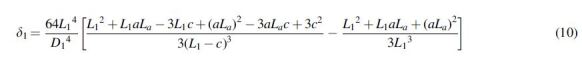



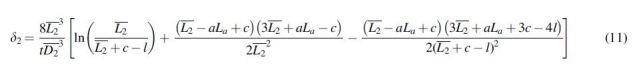


(12) $$\delta_{3}=\frac{64{L_{3}}^{4}}{{D_{3}}^{4}}\frac{{\left(aL_{a}-l\right)}^{3}}{3{L_{3}}^{3}\left(L_{3}-aL_{a}+l\right)}.$$

Consequently, Equation (8) denotes the dependence of contact deflection $\delta_{n}\left(aL_{a}\right)$ on the contact location $aL_{a}$, contact force *P*, geometrical parameters of the whisker's body, and material characteristic *E*, in which the determination of Young's modulus *E* is a difficult task. In this circumstance, we attempted to describe the ratio of the strain generated in the outer layer, which is linearly proportional to contact force and material stiffness, to the curvature of the whisker.

Hooke's law is applied to construct the relationship between bending stress σ and the strain $\delta_{n}$ measured by the sensing element as follows:

(13) $$\delta_{n}=\frac{\sigma }{E}=\frac{yM\left(x\right)}{EI_{2}\left(x\right)},$$

where *y* is radius of the outermost layer of the chamber's wall where the strain is measured:

(14) $$y=\frac{1}{2}\left[\frac{\bar{D_{2}}}{\bar{L_{2}}}\left(\bar{L_{2}}-x+c\right)+t\right].$$

Let us substitute *y*, M(x), and $I_{2}\left(x\right)$ into Equation (13) to get the final form:

(15) $$\begin{array}{cc} \delta_{n} & =\frac{4P{\bar{L_{2}}}^{3}}{E\pi t{\bar{D_{2}}}^{3}}\int_{x_{ss}}^{x_{ss}+e}\frac{\left[\frac{\bar{D_{2}}}{\bar{L_{2}}}\left(\bar{L_{2}}-x+c\right)+t\right]\left(aL_{a}-x\right)}{{\left(\bar{L_{2}}-x+c\right)}^{3}}dx\\ & =\frac{4P{\bar{L_{2}}}^{3}}{E\pi t{\bar{D_{2}}}^{3}}\kappa , \end{array}$$

where $x_{ss}$ and *e* are location and measuring grid length of the strain gauge as shown in Figure 3A, and:

(16) $$\kappa =\int_{x_{ss}}^{x_{ss}+e}\frac{\left[\frac{\bar{D_{2}}}{\bar{L_{2}}}\left(\bar{L_{2}}-x+c\right)+t\right]\left(aL_{a}-x\right)}{{\left(\bar{L_{2}}-x+c\right)}^{3}}dx$$

Combining Equations (9) and (15) yields:

(17) $$\begin{array}{cc} \frac{\delta_{n}}{\delta_{n}\left(aL_{a}\right)}=\frac{4{L_{2}}^{3}}{t{D_{2}}^{3}}\frac{\kappa }{\delta_{1}+\delta_{2}+\delta_{3}} & =f_{n}\left(aL_{a}\right)\Leftrightarrow \delta_{n}\\ & =f_{n}\left(aL_{a}\right)\times \delta_{n}\left(aL_{a}\right). \end{array}$$

Equation (17) implies that the tactile signal in the form of mechanical strain relies on the geometry, the location of the stimuli source, and the deformation of the whisker (candidates for contact force *P* and Young's modulus *E*). Validation of the above model was examined by experimental results discussed later.

### Compensation mode

The proposed whisker is able to use the compensation mode when it was trimmed or broken along its body. In this mode, the air pressure inside the medulla chamber is varied, bringing change in chamber morphology, which results in an adjustable output of the strain gauge (attached to the chamber outside wall). For the accomplishment of this purpose, the analytical model must predict outputs of the sensing element (strain gauge) for different dimensions of chamber morphology (Fig. 3B). Let us introduce variable Δl(x) representing the axial deformation at any cross-sectional area of the chamber when the inner pressure changes. With this notation, Δl(l) is the total axial deformation of the chamber, which is directly measured by a laser sensor (discussed in the experiment section). The ultimate aim is to obtain the new configuration of the whisker model after pressurization with respect to Δl(l). For ease of modeling, several assumptions were made as follows:

- Possible deformation upon pressurization of the chamber was assumed to be in region 2. As a result, the morphology of region 1 and region 3 remains unchanged under any pressure value. Extension in the length of region 2 may only be caused by pressure force *Q* (represented by black arrows in Fig. 4C) in the *x*-direction because of reinforced fibers.
- The diameter at each end of the chamber does not change during air pressurization. Region 2, in general, and the chamber, in particular, remain linear-tapered upon pressurization.
- The intersection of region 2 and region 3 was stair-like type as illustrated in Figure 3B, instead of a fillet shoulder.
- Thickness *t^q^*, corresponding to the pressure *Q* in the chamber, was considered to remain a linear distribution across region 2. Thus, *t^q^* is calculated as the average value of thickness at two arbitrary cross-sectional areas near two ends of region 2 [we chose $t^{q}\left(2.5\right)$ and $t^{q}\left(l\right)$]. We estimated that the approximation error rate was about 7.3% according to the highest examined pressure (discussed further in the experiment section).

The estimation equation for $t^{q}\left(x\right)$ is derived using the ratio of transverse strain to axial strain, which is simply represented by the Poisson's ratio coefficient υ, and calculated as follows:

(18) $$\upsilon =\frac{\frac{\Delta R_{2}\left(x\right)}{R_{2}\left(x\right)}}{\frac{\Delta l\left(l\right)}{l-c}}\Leftrightarrow \Delta R_{2}\left(x\right)=\Delta t\left(x\right)=\frac{\upsilon \Delta l\left(l\right)R_{2}\left(x\right)}{l-c}$$

(19) $$\Rightarrow t^{q}\left(x\right)=t-\Delta t\left(x\right).$$

Poisson's ratio of a typical silicone-rubber material was estimated in a tension experiment as approximately equal to 0.5^34^; in this study, we chose the value of 0.49. Then, thickness *t^q^* applying along region 2 is calculated as:

(20) $$t^{q}=\frac{t^{q}\left(2.5\right)+t^{q}\left(l\right)}{2},$$

where $t^{q}\left(2.5\right)$ and $t^{q}\left(l\right)$ are thickness of the cross section at x=2.5 mm and x=l, respectively. The next variable we need to identify is Δl(x). We applied elastic mechanic theory to derive the axial deformation Δl(x) by the expression below:

(21) $$\Delta l\left(x\right)=\frac{F}{E_{2}}\int_{c}^{x}\frac{1}{A\left(x\right)}dx,$$

in which *F* is an internal force generated on the cross-sectional area at *x* (indicated by red arrows in Fig. 4C) with area A(x). They are calculated by:

(22) $$F=Q\frac{\pi {d_{m}}^{2}}{4},$$

(23) $$\begin{array}{cc} A\left(x\right)=\frac{\pi {d_{2}}^{2}\left(x\right)}{4}-\frac{\pi {d_{m}}^{2}\left(x\right)}{4} & =\frac{\pi {D_{2}}^{2}}{4{L_{2}}^{2}}{\left(L_{2}-x+c\right)}^{2}\\ & -\frac{\pi {D_{m}}^{2}}{4{L_{m}}^{2}}{\left(L_{m}-x+c\right)}^{2}. \end{array}$$

Rewriting Equation (16), we obtained following relation:

(24) $$\begin{array}{cc} \Delta l\left(x\right) & =\frac{Q{d_{m}}^{2}{L_{2}}^{2}{L_{m}}^{2}}{E_{2}}\\ & \int_{c}^{x}\frac{1}{{D_{2}}^{2}{L_{m}}^{2}{\left(L_{2}-x+c\right)}^{2}-{D_{m}}^{2}{L_{2}}^{2}{\left(L_{m}-x+c\right)}^{2}}. \end{array}$$

By the combination of above-derived equations, relation of Δl(x) w.r.t Δl(l) can be obtained as follows:

(25) $$\Delta l\left(x\right)=\frac{\int_{c}^{x}\frac{1}{{D_{2}}^{2}{L_{m}}^{2}{\left(L_{2}-x+c\right)}^{2}-{D_{m}}^{2}{L_{2}}^{2}{\left(L_{m}-x+c\right)}^{2}}}{\int_{c}^{l}\frac{1}{{D_{2}}^{2}{L_{m}}^{2}{\left(L_{2}-x+c\right)}^{2}-{D_{m}}^{2}{L_{2}}^{2}{\left(L_{m}-x+c\right)}^{2}}}\Delta l\left(l\right).$$

Consequently, Table 2 shows all changed parameters for reconstructing the original analytical model introduced in the previous section and their new approximation equations. Otherwise, as discussed in Silicon-Rubber Characterization section, Young's modulus of region 2 (*E*_2_) is expected to vary corresponding to the change in chamber morphology with pressurization. Hereafter, this variation is described by variable *k*_2_ with the following relation: $E_{2}^{q}=k_{2}E_{2}=k_{2}E$. With new configuration as specified above, we finish reconstructing the analytical model for compensation mode by adjusting Equations (10–12) as:

**Table 2.** Reidentification of Whisker's Structure

Original param.	Param. after pressurization	Original param.	Param. after pressurization
$\bar{D_{2}}$	$\bar{{D_{2}}^{q}}=D_{m}+t^{q}$	*l*	$l^{q}=l+\Delta l\left(l\right)$
$\bar{d_{2}}$	$\bar{{d_{2}}^{q}}=d_{m}+t^{q}$	*L_a_*	${L_{a}}^{q}=L_{a}+\Delta l\left(l\right)$
$\bar{L_{2}}$	$\bar{{L_{2}}^{q}}=\frac{\bar{{D_{2}}^{q}}\left(l^{q}-c\right)}{\bar{{D_{2}}^{q}}-\bar{{d_{2}}^{q}}}$	$x_{ss}$	${x_{ss}}^{q}=x_{ss}+\Delta l\left(x_{ss}\right)$


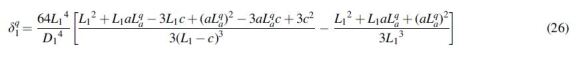



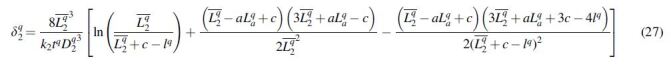


(28) $$\delta_{3}^{q}=\frac{64{L_{3}}^{4}}{{D_{3}}^{4}}\frac{{\left(aL_{a}^{q}-l^{q}\right)}^{3}}{3{L_{3}}^{3}\left(L_{3}-aL_{a}^{q}+l^{q}\right)}$$

as well as Equation (15):


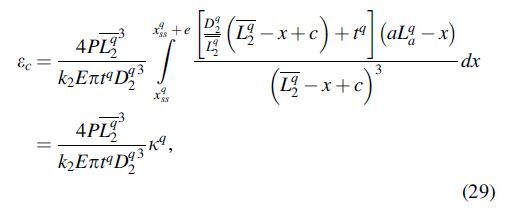


where:

(30) $$\kappa^{q}=\int_{x_{ss}^{q}}^{x_{ss}^{q}+e}\frac{\left[\frac{D_{2}^{q}}{\bar{L_{2}^{q}}}\left(\bar{L_{2}^{q}}-x+c\right)+t^{q}\right]\left(aL_{a}^{q}-x\right)}{{\left(\bar{L_{2}^{q}}-x+c\right)}^{3}}dx$$

Finally, the final equation to compute the numerical strain in the compensation mode is:

(31) $$\begin{array}{cc} \frac{\delta_{c}}{\delta \left(aL_{a}^{q}\right)}=\frac{4{\bar{L_{2}^{q}}}^{3}}{k_{2}t^{q}{\bar{D_{2}^{q}}}^{3}}\frac{\kappa^{q}}{\delta_{1}^{q}+\delta_{2}^{q}+\delta_{3}^{q}} & =f_{c}\left(aL_{a}^{q}\right)\Leftrightarrow \delta_{c}\\ & =f_{c}\left(aL_{a}^{q}\right)\times \delta \left(aL_{a}^{q}\right). \end{array}$$

## Experimental Results

### Experiment design

**FIG. 5.**
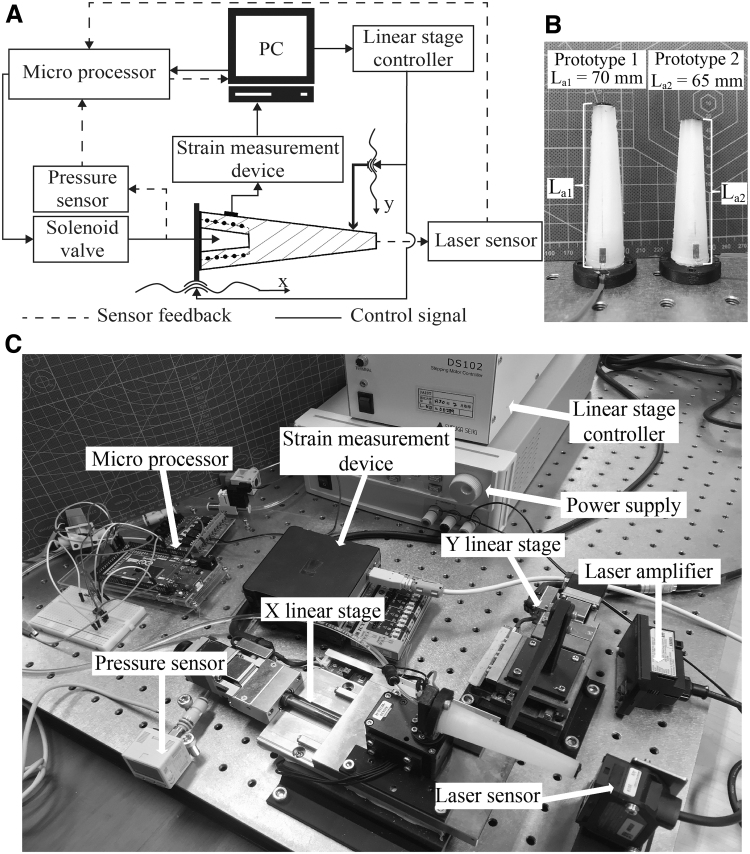
Experimental setup. **(A)** Block diagram of the experimental setup. **(B)** Experimental prototypes 1 and 2 have the identical dimensions as listed in Table 1 except the actual length $L_{a1}=70$ and $L_{a2}=65$ mm, respectively. **(C)** Experimental setup includes the data acquisition system, sensors (pressure sensor and distance sensor), and power supply.

To evaluate the reliability of the proposed sensing system, an experimental setup was designed as illustrated in Figure 5. It comprised an artificial whisker and an obstacle (with a rigid sharp edge), fixed onto two linear motorized stages (PG750-L05AG-UA; Suruga Seiki, Japan) perpendicular to each other. They were driven precisely at a resolution of 2 μm by a stepping motor controller (DS102; Suruga). The *X* linear stage was used to ensure that the contact was exerted at the desired distance from the base, whereas the *Y* linear stage drove the obstacle back and forth for expected deflection with velocity v=0.5 mm/s. In this research, we examined two prototypes of artificial whisker with equivalent structure parameters as listed in Table 1 but different length: $L_{a1}=70$ and $L_{a2}=65$ mm, to represent an intact and trimmed whisker. The strain output was measured by an instrument EDX-15A connected with a bridge box UI-54A-120 and sent to the computer for postprocessing by data acquisition program DCS-100A (these devices are from Kyowa, Electronic Instrument Co., Japan). The sampling rate for the data recording system was set at 100 Hz.

To acquire information on pressure value and axial deformation of the chamber in the compensation mode, a digital pressure sensor ISE20A-R-M (SMC Co., Japan) and an analog laser sensor IL-030 integrated with amplifier IL-1050 (Keyence Co., Japan) were used. The sensor head of the laser was fixed in a mounting bracket so that the laser light from the transmitter was coincident to the centerline of the whisker. Moreover, to ensure that the laser light absorption or excessive reflection did not affect the measurement, a black layer of silicone covered the free end of the whisker. All analog signals from these devices were recorded by a microprocessor (Arduino MEGA 2560) and transmitted to the computer. Finally, MATLAB R2019a was used for data analysis.

### Procedure for Young's modulus estimation

In this section, a static test to observe the variation of the material property of region 2 (*E*_2_) under increased air pressure in the chamber was conducted. In detail, we evaluated the response of the strain gauge generated by expansion in the *x*-direction of the chamber to compute the value of *k*_2_ ($E_{2}^{q}=k_{2}E_{2}=k_{2}E$). Note that sensitivity adjustment relied on the change in morphology of the chamber, while a typical response of an individual sensing element did not remarkably change. At first, the signal of strain gauge generated by axial stress during air pressurization can be derived as follows:

(32) $$\begin{array}{cc} \delta_{a} & =\frac{\Delta l_{ss}}{e}=\frac{Q{d_{m}}^{2}{L_{2}}^{2}{L_{m}}^{2}}{eE_{2}^{q}}\\ & \int_{x_{ss}}^{x_{ss}+e}\frac{1}{{D_{2o}}^{2}{L_{m}}^{2}{\left(L_{2o}-x+c\right)}^{2}-{D_{m}}^{2}{L_{2}}^{2}{\left(L_{m}-x+c\right)}^{2}}, \end{array}$$

where $\Delta l_{ss}$ represents for the axial deformation at the area where the strain gauge is bonded and $\delta_{a}$ is the measured strain. Then, Young's modulus of region 2 is computed by regressing strain output $\delta_{a}$ and pressure *Q*:

(33) $$\begin{array}{cc} E_{2}^{q} & =\frac{Q{d_{m}}^{2}{L_{2}}^{2}{L_{m}}^{2}}{e\delta_{a}}\\ & \int_{x_{ss}}^{x_{ss}+e}\frac{1}{{D_{2o}}^{2}{L_{m}}^{2}{\left(L_{2o}-x+c\right)}^{2}-{D_{m}}^{2}{L_{2}}^{2}{\left(L_{m}-x+c\right)}^{2}}. \end{array}$$

**FIG. 6.**
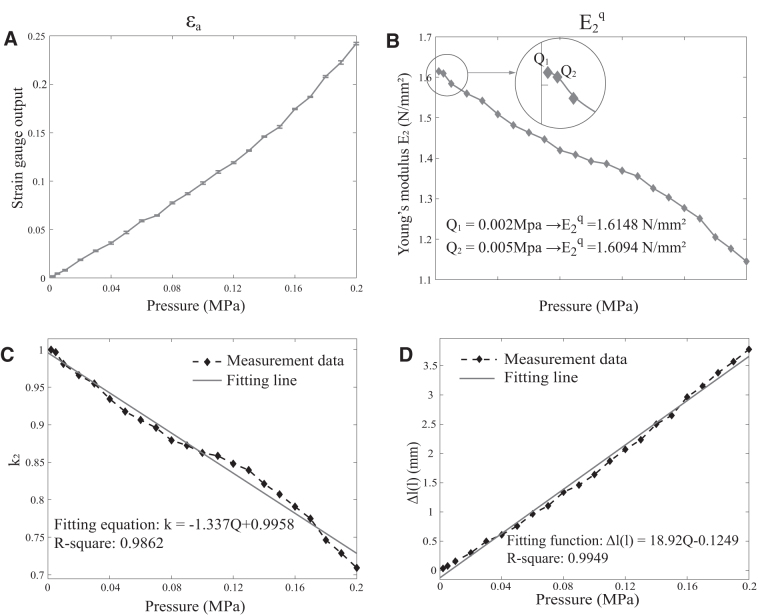
Variation of material characteristics with respect to a range of pressure *Q*: **(A)** Sensor signals with standard deviation due to the axial elongation of the chamber were recorded in five times. **(B)** Young's modulus $E_{2}^{q}$ estimation result in which, at low pressures, $E_{2}^{q}$ shows insignificant variability. **(C)** The corresponding value of *k*_2_ and the approximate function depend on the pressure *Q*. **(D)** Deformation of the whisker model in *x*-direction measured by the distance sensor.

We regulated pressure *Q* within the range 0.002÷0.2 MPa and repeatedly recorded the response of the strain gauge five times. The examined range of *Q* is decided to avoid exceeding zone B (Fig. 6C), which might increase the error of the estimation method for Young's modulus (Procedure for Young's Modulus Estimation section) or permanently ruin the original structure. The results are plotted in Figure 6A. Output in μm/m from the measuring device is converted by the equation: $\delta =\frac{\delta_{read}}{GF}\times 10^{-3}$, where $\delta_{read}$ is read from the software and GF stands for the gauge factor provided by the vendor (GF=2.21%±1%). Then, by substituting the mean values of recorded strain for each trial into Equation (33), we can derive the variation of $E_{2}^{q}$ in Figure 6B. Figure 6B illustrates a significant decreasing trend of Young's modulus corresponding to the increase of pressure inside the chamber as expected. Note that at very low chamber pressures of the chamber ($Q_{1}=0.002$ and $Q_{2}=0.005$ MPa), that is, with small deformations of the chamber, the value of $E_{2}^{q}$ slightly falls, which reminds us about the early stage of the strain–stress curve (zone A in Fig. 2C). Thus, we may set Young's modulus corresponding to pressure value $Q_{1}=0.002$ MPa as the initial Young's modulus *E* for the whole whisker. The value of *k*_2_, then, can be directly calculated and plotted in Figure 6C (note that $E_{2}^{q}=k_{2}E_{2}$). Finally, for application to the entire examined range of pressure, we used MATLAB's curve fitting tool to find out the best-fit equation as mentioned in Figure 6C.

In conclusion, the results prove our concept that variation of the inner pressure would lead to morphological change (including softness and shape) of the whisker, as well as the response of the strain gauge. This was exploited to implement the broken whisker compensation strategy in the next section.

### Validation of the analytical model

In this section, we report an experimental method for validation of the proposed analytical model for two modes (normal and compensation) and demonstration of the feasibility of using the model to predict an appropriate chamber morphology for the compensation process (of a broken whisker). Measurement of the strain gauge response with different value of pressure in the chamber (Q=0 MPa for normal mode and Q=[0,0.002,0.05,0.1,0.15,0.2] MPa for compensation mode) was recorded and then compared to the numerical ones derived from the analytical model. All experiments were conducted within the strain gauge's sensing range. Before each trial, change in length Δl(l) corresponding to an input air pressure of the chamber was measured by a laser sensor. Figure 6D shows the obtained result and the fitting function of Δl(l) w.r.t *Q*.

**FIG. 7.**
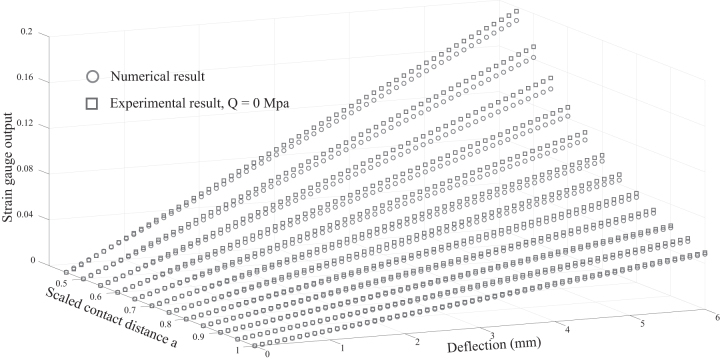
Numerical solution versus experimental result in normal mode (Q=0 MPa).

First, we validated the proposed model for the normal mode (Q=0) using prototype 1 (original whisker length $L_{a1}=70$ mm as illustrated in Fig. 5B). In this experiment, *X* and *Y* linear stages drove the whisker and the obstacle, respectively, to make contact at different locations a=0.45÷1 in steps of 0.05 along the body of the whisker and in a range of deflection $\delta \left(aL_{a}\right)=0÷6$ mm. Measurement of strain gauge output was synchronized with contact onset. The investigated ranges of contact location and deflection were chosen based on the observation that rodents tend to make subsequent touches (after an unexpected collision) in locations far from the base (along the whisker), as the whisker sweeps over an obstacle without requiring large deflection.^35^ Furthermore, any measuring inaccuracy due to exceeding the sensor threshold could be prevented. The results are analyzed and plotted in Figure 7. The output of the strain gauge, shown in Figure 7, has a quite small variance in amplitude in comparison with the numerical result, demonstrating the feasibility of the analytical model for normal mode.

**FIG. 8.**
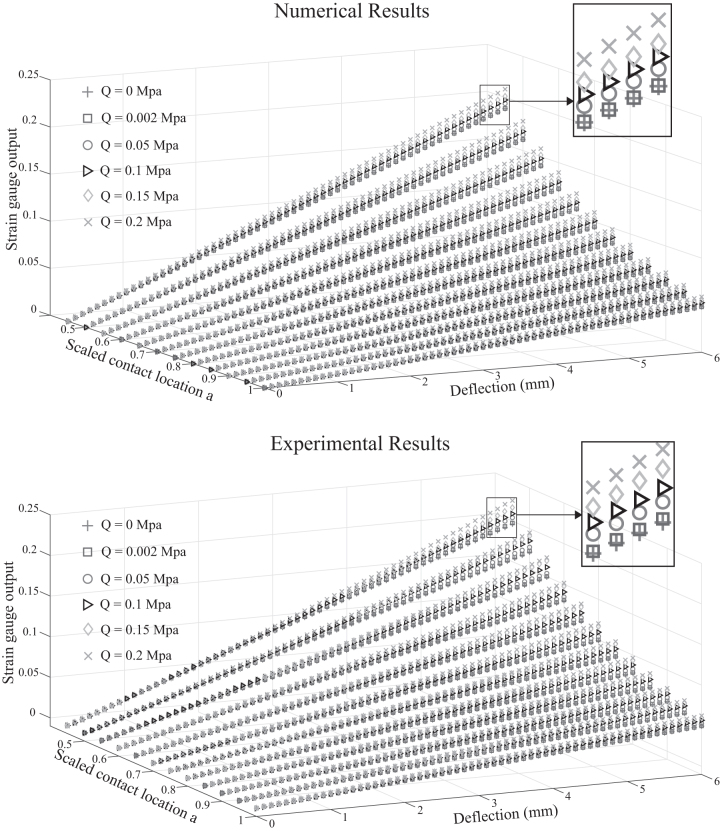
Numerical solution versus experimental result in the compensation mode: The gradient of the strain output gets higher as the input of air pressure increases.

Regarding the model in the compensation mode, a similar procedure was applied with different values of compressed air *Q* in the chamber, and the results are presented for the entire range of contact location and deflection (Fig. 8). At first glance, both graphs share a similar trend compared to the normal mode results in Figure 7, which highlights an increase in strain amplitude with pressure *Q* increase under the same interaction conditions (location and deflection). It can be explained by Figure 6B where the value of $E_{2}^{q}$ reveals a rapid decrease at high value of compressed air in the chamber, resulting in larger mechanical strain on the chamber's wall. Results in Figure 8 also implies that, by variation of inner pressure, the sensitivity of the strain gauge could be actively adjusted, which supports our original idea on the morphological change that leads to a change of sensor output and sensitivity. The blow-ups reveal that the strain output at Q=0.002 MPa is nearly coincident to that of Q=0 MPa. Hence, it is acceptable to take the value of $E_{2}^{q}$ corresponding to the pressure Q=0.002 as the initial value of the chamber's inner pressure. Accordingly, it is sufficient to keep the whisker's medulla chamber with a small value of pressure, to be more resilient to change from normal to compensation mode upon being trimmed. Based on this evaluation, the proposed whisker and the analytical model can be used as a *body* and *brain* for selection of suitable morphology to the response of different sensing tasks, especially for a compensating broken whisker.

In the next section, two case studies are examined which could emulate the compensation process inspired by rodents and demonstrate the applicability of the proposed whisker sensor to a robotic system for clarification of the efficiency of the analytical model.

### Compensation experiment

In this section, two study cases regarding a broken whisker compensated by its neighboring shorter whisker (Case 1), as well as longer whisker (Case 2), are introduced in evaluation of the suggested compensation strategy for broken whiskers. While these cases are inspired by the natural behaviors of rodents, another subcase of Case 1, which is more appropriate for a robotic system, is also investigated:

- Case 1: A short whisker compensates for a neighboring long whisker. In this case, the experimental prototype 2 (65 mm in length, Fig. 5B) plays the role of *compensator*, and the prototype 1 (70 mm in length) plays the role of *target*. This also includes a subcase when a broken whisker (the short one) compensates for *itself* after being trimmed from its original (long) state.
- Case 2: A long whisker compensates for a neighboring broken (shorter) whisker. In this case, the experimental prototype 1 (70 mm in length, Fig. 5B) plays a role of *compensator*, and the prototype 2 (65 mm in length) plays a role of *target*.

The compensation technique is based on the proposed analytical model for calculation of an appropriate value of chamber pressure *Q* so that the updated sensitivity of the *compensator* is close to that of the *target* one. The experiment procedure can be summarized in the following steps:

- First, we selected the initial air pressure $Q_{1}=Q_{2}=0.1$ MPa for prototypes 1 and 2. Then, we estimated the geometrical structure and the material characteristics caused by morphology change using equations mentioned in Compensation Mode section and parameters listed in Table 2.
- Second, from the analytical model for the compensation mode in Compensation Mode section, to obtain at the similar response of the strain gauge for the *compensator* and *target* (i.e., $\delta_{c1}$ and $\delta_{c1}$, respectively), a suitable value *k*_2_ that satisfied the concept of compensation was estimated by the following equation:

(34) $$\delta_{c1}=\delta_{c2}\Leftrightarrow f_{c}\left(aL_{a1}^{q}\right)\times \delta_{c}\left(aL_{a1}^{q}\right)=f_{c}\left(aL_{a2}^{q}\right)\times \delta_{c}\left(aL_{a2}^{q}\right).$$

In this evaluation, we chose the input of contact location a=1 (at the tip of the whiskers) and deflection $\delta \left(aL_{ai}^{q}\right)=1$ mm.

**FIG. 9.**
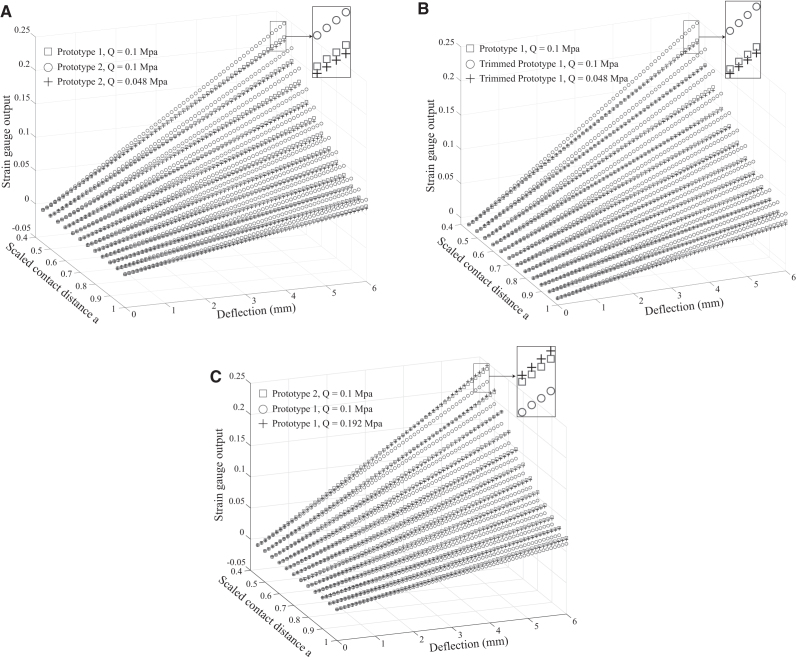
Compensation test results. Graphs in **(A, B)** are obtained strain signal at various conditions of contact location ratio and deflection of Case 1 (short whisker compensates for the neighboring long whisker) and its subcase (Shelf-compensation: a trimmed whisker compensates to itself), respectively, while graph in **(C)** is the result for Case 2 (long whisker compensates for a neighboring broken [shorter] whisker).

- Third, we substituted the value of *k*_2_ from the previous step to the relation equation between *k*_2_ and pressure *Q* to determine the necessary amount of air to be compressed into the chamber.
- Finally, the strain gauge output was recorded under different interaction conditions [a=0.45÷1 and $\delta \left(aL_{ai}^{q}\right)=0÷6$ mm (i=1,2) in steps of 0.05 and 0.1 mm, respectively] for each whisker model and is represented by blue circle markers (the original *compensator*), red square markers (the *target*), and black plus markers (the tuned *compensator*) for comparison as illustrated in Figure 9.

#### Case 1

In this scenario, the *compensator* (prototype 2) attempted to adjust its sensitivity (i.e., change pressure value *Q*) to match that of the *target* whisker (prototype 1). Substituting the required variables to solve Equation (34) reveals that the desired value of air pressure is $Q_{2}^{q}=0.048$ MPa resulting with $k_{2}=1.5033$. Simultaneously, experimental outputs of the strain gauge were recorded as shown in Figure 9A. One can observe that by decreasing the pressure from the initial value to $Q_{2}^{q}=0.048$ MPa, the response of the *compensator* decreases significantly (from blue circle markers to black cross markers) toward the *target* (red square markers) over the entire range of contact distance and deflection, regardless of a small deviation. This result guarantees that the *compensator* is able to perform similar responses in comparison with those of the *target* whisker, by solely changing pressure *Q*. We also conducted a special case for this scenario, called *self-compensation*. Specifically, prototype 1 was trimmed by 5 mm (i.e., length change from 70 mm to a value $L_{a1}=65$ mm), and then compressed air was discharged at the calculated value of 0.048 MPa. The tactile perception of prototype 1 was remeasured and compared to its own performance before being trimmed. The compensation result, as shown in Figure 9B, reveals that it is possible to adjust the sensitivity of the whisker so that it performs similarly to its own previous state (before being trimmed).

#### Case 2

In this case, the *compensator* (length $L_{a1}=70$ mm) was set to adjust its sensitivity to match the sensing performance of the neighboring *target* whisker (being trimmed with length $L_{a2}=65$ mm). To accomplish this situation, the estimated value of *k*_2_ and air pressure $Q_{1}^{q}$ were ∼0.7365 and 0.192 MPa, respectively. Then, the response of the *compensator* whisker under various contact locations and deflections was recorded and illustrated in Figure 9C. One can see that by increasing chamber pressure from its initial value to the estimated one (0.192 MPa), the compensated response of the *compensator* (black plus markers) was set to leave its original response (blue circle markers) toward and match the response of the *target* whisker (red square markers). Again, by a change in chamber morphology (by internal pressure), whisker sensing performance can be actively adjusted, demonstrating morphological compensation in tactile sensing.

In conclusion, by evaluation of two showcases on morphological compensation, despite a small difference among calculated and experimental values, the proposed whisker could actively adjust its sensitivity to match sensing performance of itself (or a neighboring whisker) when the body length shortened (trimmed or broken). At the same time, the proposed whisker design and compensation strategy can be exploited to develop *active* robotic whiskers that can actively change their sensitivity to match various sensing tasks or even to compensate itself when it is accidentally broken.

## Discussion

### Artificial whisker design with changeable morphology

The design of our artificial whisker was inspired by the structure of a rodent whisker but has some differences. The performance of the sensing element (strain gauge) is highly dependent on the morphology of the chamber layer, which differs from a natural whisker in proportion (as illustrated in Fig. 2A, B). In detail, the conical-shaped medulla of a natural whisker extends further toward the tip than the chamber in our artificial whisker, which provides different levels of elasticity. Generally, we kept the chamber's length at around 15%÷25% of the overall length for two main reasons. First, the area of the small end of the chamber was kept as large as possible to produce more internal force *F* with a little compressed air *Q* (Fig. 4C). Second, we tried to avoid physical interaction between the chamber body (region 2) and the surrounding environment, which might result in complications in deriving the numerical solutions. In addition, the damaged zone (broken, trimmed) must not be *within* the length of the region 2, otherwise, the calculations for the artificial whisker become invalid.

In contrast, the precision of the morphology analysis relies on the stable expansion of the chamber in the direction of measurement by the strain gauge, thanks to the reinforcement fiber. However, in experimental trials, a slight bend of the chamber was observed at high inner pressure. Due to this observation, the resulting strain output shown in Figure 6A may include not only pure axial translation. However, this may little affect whisker performance as long as the screw pitch *r* (Fig. 3A) of the helical path is sufficiently small. In addition, we ignored any possible impact of the nylon fiber in the analytical model and evaluation test of the material characteristics, even though they were assembled without twisting or tightening.

### Applicability: haptic sensing system for autonomous robots

A changeable morphology whisker is considered appropriate for embodied intelligence^20^ and behavior adaptivity. Our aim was to provide an efficient solution to active sensing, which allows us to actively reconfigure the sensing components to fulfill specific purposes or adapt to critical uncertainty (e.g., partly-broken state). In this discussion, we recommend two types of application configurations for our whisker-based system.

#### Type 1: an array of whiskers

An array of whiskers is capable of tactile exploration since all whisker tips lie in a plane such that an obstacle will be swept over by multiple whiskers to obtain rich information. This case deploys the biological compensation procedure discussed in Idea section, which is summarized as follows: the broken whisker will be completely and functionally replaced by its neighboring whiskers in an array of whiskers. Furthermore, whiskers in this system would require the ability to compensate for either longer (Case 1) or shorter (Case 2), which was validated by two experiment cases in Compensation Experiment section. Hence, stability is highly required for both working states. We will demonstrate how this configuration hardly meets such criteria in the next section.

#### Type 2: a single whisker

For a sole whisker system (or more than one located independently), to deal with unexpected situations (e.g., broken or trimmed whisker), it needs to be able to self-calibrate its performance. The experimental results, discussed in the previous section (Fig. 9B), demonstrate the potential of our whisker system for such application. Obviously, only air decompression is sufficient in this case. Nevertheless, the robot must be able to acquire feedback about the new configuration of the whisker after trimming to select an appropriate chamber morphology for exact compensation. This feature will be further examined in the next study. Furthermore, along with the investigation of sensing ability, we target this sensing device as a new communication protocol that a mobile robot can use to send a *message* to their allies in a swarm through *touching*, whereas the content of the message depends on the morphology of the chamber.

### Reliability of the analysis model

In this article, a numerical model of strain energy stored in each section of an artificial whisker body was introduced to analyze the consequences of morphology change if its internal chamber is changed. The convenience of this approach lies in the independent expression of the material characteristic for each region, which is significantly important for the nonuniform structure proposed in this study. Nevertheless, due to the fact that the inherent relationship between the actuation and the morphological (including geometrical and material) properties was approximated (by equations as introduced in Analytical Model for Normal and Compensation Mode and Experimental Results sections), we might be unable to obtain an explicit solution for Equation (34). Among those equations, the expression of the second moment of cross-section area for region 2, which is approximated by Equation (6), is considered to be a major factor. We compared the approximated expression $I_{2}\left(x\right)$ with an exact one and then calculated the maximum approximation error which ranged from 4.7% to 11.51% with chamber width (Fig. 3) from 33% to 50% of the outer radius of the large end. Thus, to minimize any error in the analytical model, the value of *t* should be set as small as possible with regard to the possibility of the fabrication process.

In contrast, quasi-static tests to observe responses of the embedded sensing element under different contact conditions were conducted with a wide range of chamber morphology. Comparison results in Procedure for Young's Modulus Estimation and Validation of the Analytical Model sections show consistency with the hypothesis we made at the beginning of this study. The first agreement is related to the decreasing trend of Young's modulus *E*_2_ when the air pressure in the chamber increases (as illustrated in Fig. 6B), which conforms to the stress–strain curve (Fig. 2C). The second agreement is that the experimental results of the analytical model could predict the overall performance of the system. Thus, the model could be utilized to serve as a *brain* to actively control the whisker *body* depending on various tasks such as adaptive functions.

Although the performance of the whisker prototypes in compensation mode (Fig. 9) tended to agree with the hypothesis, some challenges still remain. Some errors in the measurement data may have arisen during analog-to-digital conversion by the microprocessor system or the proposed analytical model itself does not fully consider all potential factors affecting system performance. For overall evaluation, we examined how the parameters of actual length *L_a_* and air pressure *Q* influenced the *compensator* performance in the compensation mode. Moreover, we evaluated how accuracy of the proposed model changed in compensation mode, when the whisker was trimmed at different locations. An experimental procedure similar to that introduced in Compensation Experiment section was conducted on the *compensator* whisker with the length varied from 65 to 69 mm in Case 1 and 66 mm to 70 mm in Case 2. Under each condition of trimmed whisker, the desired inner pressure *Q* was estimated from the model and applied to the chamber. Then, the difference between the strain signal of *compensator* whisker and its *target* one after implementing the compensation was computed through Equation (35).

(35) $$Compensationerror\left(\%\right)=\left|\frac{\delta_{ac}-\delta_{t}}{\delta_{bc}-\delta_{t}}\right|\times 100,$$

where $\delta_{ac}$, $\delta_{bc}$, and $\delta_{t}$ are tactile responses of *compensators* after and before compensation process and its target, respectively. In short, the smaller the compensation error is, the more accurate the compensation process is.

**FIG. 10.**
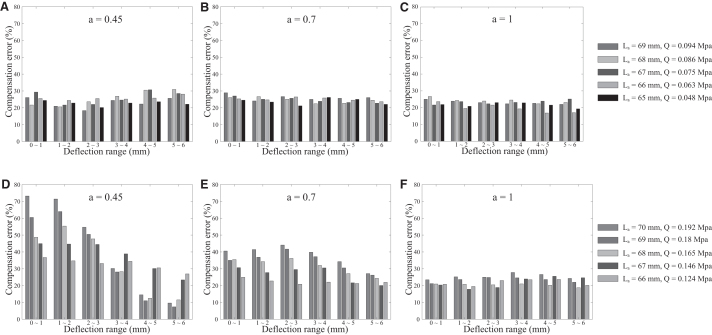
Compensation error analysis for Case 1 **(A–C)** and Case 2 **(D–F)** with different actual length (*L_a_*) and chamber pressure (*Q^q^*) of *compensator*. The contact location ratio *a* was varied from 0.45 (contact location is near the tip of the chamber), 0.7 (contact location is near the center of the trimmed whisker), and 1 (contact location is at the tip of the trimmed whisker), while the pressure *Q^q^* was estimated from the analytical model. Error was calculated based on experimental results as shown in Equation (35).

Figure 10 compares the average compensation error with respect to a specific range of deflection at three different contact locations, that is, the contact location ratio *a* was varied from 0.45 (contact location is near the tip of the chamber), 0.7 (contact location is near the center of the trimmed whisker), and 1 (contact location is at the tip of the trimmed whisker). Generally, the error of Case 1 (Fig. 10A–C) was rather consistent under most conditions of length and pressure and smaller than most conditions of error of Case 2 (Fig. 10D–F), especially when contact occurred near the tip of the chamber (i.e., a=0.45). The factors affecting the overall accuracy of Case 2 can be characterized as below:

- Figure 10D illustrates that, at the early stage of whisker bending (very small deflection), the larger the value of the chamber inner pressure, the larger the compensation error. This can be explained by the fact that at high pressure, the chamber is stretched resulting in a significant increase of tension of the chamber wall, which requires greater contact force even with small deflection. As a result, the outer surface of region 2 experienced a *local deformation* at the interacted region, leading to an amount of strain output absorbed.
- Whereas, at higher deflection ranges, the bending stress increases enormously producing larger mechanical strain output complying with the stress–strain curve in Figure 2C. Thus, the compensation error decreases dramatically which can be observed more clearly for the high-pressure cases (e.g., blue and orange columns). As can be seen from Figure 10E and F, the influence of such factors described above was no longer significant due to the inversely proportional relation between contact location and contact force.

For other cases (Fig. 10A–C), the results were consistent with the above arguments. Generally, the greatest error occurred with contact close to the base (a=0.45). However, there was no remarkable difference compared with the other contact location since the air compressed in the chamber was relatively small for all testers.

According to above investigation, we conclude that, with the morphology of the artificial whisker suggested by the analytical model, the *compensator* in Case 1 accomplished the compensation task with more stability and lower overall average error (20.385%) for the whole range of contact locations and deflections, compared to Case 2 (36.1837%). This suggests that it is better to configure the proposed device according to type 2, whose chamber pressure is always lower than the initial value in compensation mode, rather than type 1.

### Affecting factors of compensable range

In the proposed whisker sensory system, the largest compensable range of a shortened whisker is considered as a fundamental feature of the compensation ability. This feature is also understood as the maximum and minimum level of tactile perception that a whisker can actively adjust to accomplish compensation tasks with an acceptable accuracy rate. According to the compensation principle clarified in Idea and Analytical Model for Normal and Compensation Mode sections, this range is strongly dependent on the morphological transformation of the chamber. In this study, we tested two scenarios (Compensation Experiment section) with trimmed length up to 5 mm. However, it should be noted that the proposed compensation process also works for a wider range. To tackle this issue, the value of the initial air pressure *Q_i_* (*i* = 1, 2) assigned in the experiment Compensation Experiment section need to be altered to 0.2 MPa for Case 1 and 0 MPa for Case 2, respectively. As a result, the changeable range of air pressure inside the chamber, which is proportional to the morphological transformation level, will be increased accordingly.

Nevertheless, increasing the adjustable range of the compressed air inside the chamber to claim a better compensation range is not always advantageous due to a higher resulted compensation error (as pointed out in Reliability of the Analysis Model section). Furthermore, in such attempt, the air regulating system needs a more power-consuming and bulkier compressor, which is not suitable especially for a small-scale robotic system. An alternative approach is to optimize the geometrical variables of the whisker body especially the chamber such as the chamber length *l*, the thickness *t*, and outer diameters of each region (Fig. 3) to achieve the maximum compensable range (upon application's demand) with a small value of compressed air. Since such approach requires more thorough investigation on the correlation among influencing elements to the compensation range, it remains in our future work as a comprehensive fulfillment of the idea of morphology-based tactile compensation.

## Conclusion

The novelty of this artificial vibrissa sensing device is the variable morphology of the medulla-like chamber actively controlled by an external pneumatic actuator to accomplish the recalibration task for the tactile perception of a broken or trimmed whisker. In the compensation mode, all potentially deformable parameters were considered in the analytical model and estimated with respect to the inner pressure of the chamber. In addition, various experiments confirmed that the material characteristic reflected in the term Young's modulus *E* was strongly dependent on the morphology of the chamber. This indicates that the sensitivity of the sensing element (strain gauge) can be controlled using the analytical model. In addition, the feasibility and variability of the proposed device were investigated through two separate experimental cases, in which Case 1 (a short whisker compensating for a longer one or self-compensation) outperformed its counterpart with only 20.385% compensation error. Furthermore, to our knowledge, this is the first whisker design for a robotic system that incorporates the medulla layer to achieve tactile perception.

In future work, we will explore new methods to optimize the design and increase its precision rate. The analytical model based on the morphological computation technique will be investigated more thoroughly to exploit this idea for other applications such as a failure warning system. In addition, the efficiency of this sensory system will be verified by integrating it into a robotic system.
